# Supplementary material for: Chlorhexidine cord care after a national scale-up as a newborn survival strategy: A survey in four regions of Ethiopia
Source: PLoS One. 2022 Aug 5;17(8):e0271558. doi: 10.1371/journal.pone.0271558 (PMC9355194; doi:10.1371/journal.pone.0271558)
Supplement: Attachment — Submitted filename: Response to Reviewers.docx [file pone.0271558.s002.docx]

Point-by-point response to reviewers’ comments

**********************************************************************************

**Reviewer #1:**

**Comment:** This study titled, "Chlorhexidine cord care after a national scale-up as a newborn survival strategy: A survey in four regions of Ethiopia" assess the use of Chlorehexidine to umbilical cord care in 4 regions of Ethiopia. The study is well designed and the methods are well described. The results are clearly presented and the conclusions are appropriate. I have no major comments about the study.

**Response:** Thank you for your kind and positive assessment of our manuscript.

**Reviewer #2:**

**Comment:** The authors in this paper have attempted to assess the coverage and determinants of an evidence based intervention for reducing newborn morbidity and mortality in a high burden setting. However, I have the following major concerns with the study methods and rigor:

1. My main concern with the paper is that the objective of the study was not to assess the coverage and determinants of Chlorhexidine use after a national scale-up, but as the authors state in the paper that the survey was conducted as a part of the baseline assessment for the "Nutrition International’s (NI’s) Maternal, Newborn Health and Nutrition (MNHN) Programme planned to be implemented in the survey districts over 2020-2024. Promoting the use of Chlorhexidine for cord care is among the package of services included in the NI’s MNHN programme." Therefore, the variables assessed as determinants are the ones that were available from the survey findings rather than the potential determinants of the Chlorhexidine use. As a result, the association has only been determined with a handful of available variables (including maternal education, region of residence, skilled attendance during the last birth, and maternal knowledge of the benefit of chlorhexidine cord care). There are many other potential variables to be explored in the model that could impact the coverage including implementation coverage of national scale-up, healthcare provider, availability of chlorhexidine, healthcare worker knowledge etc. Without information of these critical variables, the findings stand incomplete.

**Response:** Thank you for raising this concern. As you correctly pointed out, the present work is part of a larger survey designed to address various maternal and newborn health and nutrition (MNHN) variables. The data on the use of chlorhexidine for cord care and its determinants was obtained from the mentioned larger survey. As pointed out, the data used lacks some important variables that could affect use of chlorhexidine for cord care. We have recognized this fact and addressed the issue in the discussion section of the paper. In the third paragraph of the discussion section (page 23, lines 359-362), we have argued that the variation in the coverage of chlorhexidine use for cord care across regions may be attributable to variations in the strength of the programme implementation and follow up. In that argument we are implying that even though the strength/intensity of the programme implementation was not measured and analyzed as a determinant, it could still be playing a role by operating through other variables such as region of residence. Further, in the sixth paragraph of the discussion section (pages 24-25, lines 389-393), we have discussed as a limitation of the study that it did not address all important determinants of chlorhexidine use for cord care. We especially noted that “supply-side determinants such as availability of sufficient chlorhexidine stock, and health workers’ knowledge of and willingness to provide chlorhexidine cord care were not included.” We feel that, given such information, readers can interpret the results accordingly.

**Comment:** 2. My second concern with the study is self-reporting of chlorhexidine use. Considering that the sample included mothers 0-11 months post partum, I suspect that the findings are highly prone to reporting and recall bias.

**Response:** Thank you for this concern as well. We also have recognized the possibility of recall bias and have stated it as part of the limitation of the study in the sixth paragraph of the discussion section (pages 24-25, lines 393-395). Furthermore, in the Materials and Methods section, under Variables of the Study (page 9, lines 169-171), we have stated that “A coloured picture of chlorhexidine tube was shown to the mothers to help them recall what chlorhexidine is.” Therefore, both what has been done to minimize the possibility of recall bias and the limitation of the study vis-à-vis recall bias have been addressed in the paper.

**Comment:** My suggestion to the authors would be to re-write this paper as a baseline survey findings for the "Nutrition International’s (NI’s) Maternal, Newborn Health and Nutrition (MNHN) Programme" and report all the variables assessed rather than report as a study targeted to explore the coverage and determinants, which would be scientifically more rigorous and logical.

**Response:** Thank you for the suggestion. The MNHN survey is a large survey addressing several maternal and newborn health and nutrition indicators. The results of that large survey couldn’t be condensed into a single journal article. Besides, of the various indicators addressed by the MNHN survey, a pronounced knowledge gap exists on the use of chlorhexidine for cord care and its determinants. We therefore aimed to contribute to the meagre literature pool on chlorhexidine use for cord care. Thus, this paper which focused on chlorhexidine use would be worthy in terms of conveying more needed information on an issue not sufficiently researched before.

**Comment:** Some specific comments below:
Abstract:
In the abstract 'methods' section, the authors have provided the year when national scale-up was implemented. I would suggest that the authors also provide the year of the survey for context.

**Response:** Thank you for this comment. We now have included the survey period in the methods part of the abstract (page 2, line 29).

**Comment:** Introduction:
Correct line 65: "Ethiopia is one of the five countries in which half OF all the global under-five deaths occur"

**Response:** Thank you for pointing this out. Corrected accordingly (page 4, lines 64-65).

**Comment:** Methods:
Under study setting, the authors quote "As per the 2007 National Housing Census of Ethiopia, these four regions cover more than 85% of the national population". I would suggest the authors replace this with updated figures, if available, as it seems too old.

**Response:** Thank you for this concern. The last census in Ethiopia was conducted in 2007. Any description of the population profile and population composition of Ethiopia is based on the 2007 census and projections emanating from it. We are therefore retaining the description which is based on the 2007 census as it was.

**Comment:** Adding a geographical map in this section might help visualize the study settings for the readers more than the text.

**Response:** Thank you for this suggestion. We now have included a map which distinguishes regions covered by the survey and those not covered by the survey. Please see Fig 1, which is also cited within text on page 7.

**Comment:** Under 'sample size and sampling', the authors state that "As this study was part of a larger survey, sample size was estimated to ensure that it would be sufficient to estimate all key indicators covered by the NI-supported MNHN programme." Although the authors state that the sample size was sufficient to achieve the objectives of the present study and analyze the determinants of chlorhexidine cord care, I would suggest that the authors specify which variables have they considered as determinants for the sample size assumptions.

**Response:** Thank you for this comment. We did a post-hoc analysis of the adequacy of the sample size to analyze determinants. Accordingly, we took the results on the determinants of chlorhexidine cord care reported in the presented paper and estimated the minimum detectable effect sizes in terms of odds ratios. We now have revised the description to make the message clearer (page 8-9, lines 148-154).

**Comment:** Correct line 199-200: "The present STUDY analyses utilized data from both the socio-economic module and the MNHN module."

**Response:** Thank you for this concern. We found the original description to be more proper than the suggested one. Therefore, we retained the original sentence.

**Comment:** Results:
The confidence intervals for the associations between chlorhexidine use and 'skilled birth attendance' and 'knowledge about benefit' is too wide signifying the points made earlier.

**Response:** Yes, thank you for noting this. The 95% confidence intervals (CIs) of the adjusted odds ratios for 'skilled birth attendance' and 'knowledge about benefit' are very wide. Yet, looking even at the lower limit of the CIs, it is notable that the magnitude of association of those variables with the use of chlorhexidine core care is large.

**Comment:** Discussion:
The authors have rightly pointed out in the discussion section that some of the variables found to be associated with the use of chlorhexidine are merely chance findings or a results of reverse causality for example skilled birth attendance and knowledge of benefits of chlorhexidine.

**Response:** Thank you for noting this. Yes, we have provided possible and plausible explanations for the observed associations.
