# Supplementary material for: Chlorhexidine cord care after a national scale-up as a newborn survival strategy: A survey in four regions of Ethiopia
Source: PLoS One. 2022 Aug 5;17(8):e0271558. doi: 10.1371/journal.pone.0271558 (PMC9355194; doi:10.1371/journal.pone.0271558)
Supplement: Attachment — Submitted filename: Response to Reviewers.docx [file pone.0271558.s003.docx]

Rebuttal letter (point-by-point response to journal requirements and reviewers’ comments)

**********************************************************************************

**Journal requirements**

**Requirement:** Please review your reference list to ensure that it is complete and correct. If you have cited papers that have been retracted, please include the rationale for doing so in the manuscript text, or remove these references and replace them with relevant current references. Any changes to the reference list should be mentioned in the rebuttal letter that accompanies your revised manuscript. If you need to cite a retracted article, indicate the article’s retracted status in the References list and also include a citation and full reference for the retraction notice.

**Response:** We have carefully reviewed all articles included in the reference list. All references are correct and complete. None of the articles in the reference list is a retracted one as of the date of writing this response.

**Reviewer #3:**

**Comment:** Though the authors have addressed all the comments, but I still believe the comment from Reviewer #2 Comment #1 has not been addressed sufficiently. I agree with reviewer that many important variables have been ignored and therefore the model stands incomplete. How do authors think these results should be interpreted when they also agree to this issue

**Response:** Thank you for raising this point again. As you pointed out, this was a critical comment raised by Reviewer #2 during the first round of review. Accordingly, we have considered the comment duly, and provided an elaborate response with which Reviewer #2 agreed.

To reiterate our response to Reviewer #2’s comment during the first round of review, ‘… the present work is part of a larger survey designed to address various maternal and newborn health and nutrition (MNHN) variables. The data on the use of chlorhexidine for cord care and its determinants was obtained from the mentioned larger survey. As pointed out, the data used lacks some important variables that could affect use of chlorhexidine for cord care. We have recognized this fact and addressed the issue in the discussion section of the paper. In the third paragraph of the discussion section (page 23, lines 359-362), we have argued that the variation in the coverage of chlorhexidine use for cord care across regions may be attributable to variations in the strength of the programme implementation and follow up. In that argument we are implying that even though the strength/intensity of the programme implementation was not measured and analyzed as a determinant, it could still be playing a role by operating through other variables such as region of residence. Further, in the sixth paragraph of the discussion section (pages 24-25, lines 389-393), we have discussed as a limitation of the study that it did not address all important determinants of chlorhexidine use for cord care. We especially noted that “supply-side determinants such as availability of sufficient chlorhexidine stock, and health workers’ knowledge of and willingness to provide chlorhexidine cord care were not included.” We feel that, given such information, readers can interpret the results accordingly.’

As indicated in the response to Reviewer #2 during the first round of review, we have provided context in the discussion section vis-à-vis variables not addressed in the present study. We have elaborated the implications of and limitations related to variables not included in the study. Such information would enable readers to interpret the results accordingly. We hope that Reviewer #3 would also agree with this justification.
